# Correction to ‘Chromosomal integrons are genetically and functionally isolated units of genomes’

**DOI:** 10.1093/nar/gkaf196

**Published:** 2025-03-07

This is a correction to: Paula Blanco *et al.*, Chromosomal integrons are genetically and functionally isolated units of genomes, *Nucleic Acids Research*, Volume 52, Issue 20, 11 November 2024, Pages 12565–12581, https://doi.org/10.1093/nar/gkae866


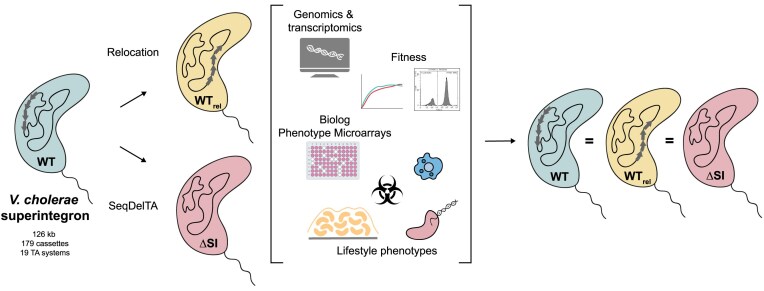


In the version originally published, there was an issue by which the genetic content of bacteria in the right side of the graphical abstract was different from the content of those to the left. Modifications have been made so that the genetic content of bacteria in the left and right parts of the graph match and are coherent.

